# Fever of Unknown Origin in a Patient with Confirmed West Nile Virus Meningoencephalitis

**DOI:** 10.1155/2014/120709

**Published:** 2014-12-14

**Authors:** Alexander Sabre, Laurie Farricielli

**Affiliations:** ^1^Universidad Autónoma de Guadalajara, Av Patria 1201, Lomas del Valle, 45129 Zapopan, JAl, Mexico; ^2^Scottsdale Healthcare Shea Medical Center, 9003 E. Shea Boulevard, Scottsdale, AZ 85260, USA

## Abstract

West Nile Virus (WNV), an RNA arbovirus and member of the Japanese encephalitis virus antigenic complex, causes a wide range of clinical symptoms, from asymptomatic to encephalitis and meningitis. Nearly all human infections of WNV are due to mosquito bites with birds being the primary amplifying hosts. Advanced age is the most important risk factor for neurological disease leading most often to poor prognosis in those afflicted. We report a case of WNV meningoencephalitis in a 93-year-old Caucasian male who presented with fever of unknown origin (FUO) and nuchal rigidity that rapidly decompensated within 24 h to a persistent altered mental state during inpatient stay. The patient's ELISA antibody titers confirmed pathogenesis of disease by WNV; he given supportive measures and advanced to an excellent recovery. In regard to the approach of FUO, it is important to remain impartial yet insightful to all elements when determining pathogenesis in atypical presentation.

## 1. Case Report

A 93-year-old Caucasian male presented to the emergency department of an urban hospital in Arizona during August with FUO, chronic neck pain, and stiffness. History of present illness is significant for a hospital admission one month prior with admitting diagnosis of FUO and dehydration. Past blood cultures of one-day duration failed to show a bacterial origin; at the time the patient was treated inpatient with IV ceftriaxone and supportive care to stabilize the patient. The patient was discharged with systemic inflammatory response syndrome (SIRS) of unknown etiology and treated outpatient with a course of clindamycin. Past medical history is significant for SIRS of unknown etiology, hypertension, acute renal failure, and osteoarthritis of the neck and knee.

The patient past surgical history is significant for cholecystectomy and tonsillectomy. Family history was noncontributory; social history revealed he was a retired veteran who lived at home with his wife. He denied recent travel and denied tobacco or alcohol use. His allergies were significant for penicillin and the patient immunizations were up to date. The patient review of systems was negative except for fevers, chills, dizziness, constipation, neck pain, stiffness, and left knee crepitus with occasional joint pain and decreased range of motion.

On admission, vital signs were a blood pressure (BP) 146/63 mmHg, heart rate (HR) 104/min, respiratory rate (RR) 20/min, temperature of 39.05°C, and oxygen saturation of 93%. Physical examination revealed numerous oral ulcers, sinus tachycardia, a 2/6 systolic ejection murmur, crepitus in the left knee, blood in urine secondary to traumatic Foley insertion, no skin rashes, and no shortness of breath. Lab values showed a procalcitonin level of <0.05 ng/mL (normal values are 0.05 ng/mL and below) and serial blood cultures were negative. An echocardiogram to rule out endocarditis was negative.

Within 24 h after admission, the patient lapsed into altered mental status (AMS), persistent fevers, nuchal rigidity, myoclonic tremors of the upper extremities, and unresponsive pinpoint pupils, along with present Babinski and Brudzinski signs (Video 1 available online at http://dx.doi.org/10.1155/2014/120709). A thorough evaluation was commenced to determine the cause of meningitis and as cause of infection was unknown, empirical treatment was begun with broad antibiotics for bacterial and Herpes simplex virus-derived meningitis, consisting of IV vancomycin, IV ceftriaxone, and IV acyclovir.

Lumbar puncture (LP) was scheduled immediately with CT of neck and head to follow. The patient's wife initially was a barrier to testing, as she believed the current state of the patient was due to traumatic Foley insertion. Informing the patient's wife of the necessity to perform this procedure was successful. Cerebral spinal fluid (CSF) results revealed clear colorless fluid with WBC of 530 *μ*L (76% neutrophils, 10% lymphocytes, and 14% monocytes); RBC of 15 *μ*L; CSF glucose of 91 mg/dL; CSF protein of 154 mg/dL. The concurrent CT of head and neck was negative. MRI showed mild spinal cord edema but did not reveal any abscesses. Culture of the CSF was VDRL negative, cocci IGG/IGM negative, HSV-1/2 PCR negative, Crypto Ag negative, Gram stain/culture negative, and fungus negative. The results of testing are shown in Table 1.

From inpatient day 1 to day 4, the patient remained in AMS, with palliative care consoled on day 3 as poor outcome of the patient was to be believed. Due to aseptic LP presentation, bacterial origin was ruled out and viral etiology was considered. After getting negative HSV ELISA titers, the differential switched to other viral diseases which can be neuroinvasive. On inpatient day 4 ELISA titers from original LP were tested and were positive for WNV, with IGG antibodies value 1.86 and IGM antibodies 4.43. Reference ranges for WNV titer were IgG < 1.30, IgM < 0.90.

Once diagnosis of WNV neuroinvasive disease was made, all IV antibiotics and antivirals were discontinued and only supportive care consisting of percutaneous endoscopic gastrostomy (PEG) tube supplemental nutrition and normal saline IV fluid was continued.

Miraculously, on inpatient day 5, the patient opened his eyes and could follow simple commands such as squeezing hands on command, waving, and speaking few words when talking with him. The myoclonic hand tremors disappeared at this time and Brudzinski sign was not present. On day 7 the patient was fully arouse-able, could speak complete sentences, and did not present with Babinski sign. From day 7 onwards the patient continuously progressed until he was transferred to skilled nursing facility on day 10 for outpatient care.

## 2. Discussion

West Nile Virus (WNV) has produced 3 of the largest arboviral neuroinvasive disease (encompassing acute flaccid paralysis, encephalitis, or meningitis) outbreaks ever recorded in the United States, with persistently high number of cases up to 3000 in 2002, 2003, and 2012 [1]. Case fatality rates in patient populations with neuroinvasive disease are approximately 10% [2]. Advanced age is the most important risk factor for death, with literature ranging from 0.8% in ages below 40 years to 17% in those aged at least 70 years [2]. Of particular interest, there is a 2-to-3-fold increase in long-term, all-cause mortality in patients discharged from hospital following acute WNV infection compared with age-adjusted population norms [3].

WNV was first isolated in a patient blood sample in the West Nile province of Uganda in 1937 [4]. Disease in humans is mainly transmitted via the infected saliva of the* Culex* mosquito species during feeding [5]. Humans, horses, and other vertebrates serve as “incidental hosts” based on low viremia stats [6]. Most infections of WNV are associated with minor “flu-like symptoms” in the majority of patients. In those patients who develop neuroinvasive disease, symptoms follow a wide spectrum from mild to severe, encompassing encephalitis, nonspecific meningitis, and asymmetrical paralysis. There is noted dichotomy in literature between populations and symptoms based on age; individuals >65 years commonly reported encephalitis compared to meningitis in patients <65 years [7]. There are also alarming outcomes based on patients with increased age. In elderly patients who develop meningitis with encephalitis, it is correlated with an approximately 10% mortality [8].

Illness in neuroinvasive disease can last for weeks to months, with long-term functional and cognitive difficulties common in patients. Extrapyramidal disorders are also infrequently observed in neuroinvasive WNV. Features of Parkinsonism may also be seen, such as the development of course tremor, which tends to be postural and contains kinetic component [9–13]. Myoclonic tremors of the upper extremities, which are present in the case, are a unique outcome of the neurovirulent nature of the virus.

The diagnostic approach is important for clinicians to adequately assess the patient in order to ameliorate causes of disease. FUO is one such patient presentation that necessitates a thorough work-up in order to find the origin of disease process. Definition of FUO includes a persistent temperature of 38.28°C lasting longer than 3 weeks; the fever has no obvious source cause despite 1-week investigation including inpatient and outpatient work-up [14]. Differential diagnosis includes infection commonly by subacute bacterial endocarditis (SBE), tuberculosis, and abdominal/pelvic abscesses. Other less common causes include malignancies, autoimmune disorders, and drug-induced FUO. Meningitis of viral origin is a diagnosis of exclusion, most often encountered after work-up for bacterial cause is ruled out. Aseptic presentation of LPs from negative Gram stain and concurrent studies rule out bacterial and fungal origin and make a viral cause of meningitis much more likely. Initial therapy before a definitive diagnosis can be made is through an empirical approach to meningitis, consisting of vancomycin, ceftriaxone, and acyclovir. By intense investigative judgement in part of astute physician practice, the origin of the FUO was found to be of infectious origin by serology titers of WNV.

Investigation of the origins of the patient disease looked at extraneous variables as cause of connection. The Arizona Department of Health Services Office of Infectious Disease Services reports the presence of WNV arbovirus statistics that showed 155 positive mosquito samples in the 2013 year with peak incidence in the late summer early fall [15]. This activity is present in the area of Arizona where the patient lives particularly with 52 confirmed and probable cases of WNV in Maricopa County [15]. When we spoke to the patient's wife about possible sources of mosquitos or open water sources, she mentioned an adjacent neighbor to their home with an uncleaned pool for an extended period of time noting on multiple occasions mosquitos outside. Although these mosquitos were not tested to prove a source of the virus, this information helped to elucidate a possible source of the disease vector.

Meningitis of viral origin is usually a diagnosis of exclusion in patients and requires astute clinical practice in order to elucidate a diagnosis. Arbovirus and concurrently WNV are known in literature to cause wide range of clinical symptoms including fever and myalgia, aseptic meningitis/encephalitis, arthritis, and a maculopapular rash. WNV encephalitis can present in many fascinating and unusual ways which may not be known to clinicians. In this case, we wish to highlight this unique presentation which involved an important lesson in clinical practice that involved multiple disciplines in order to educate health professionals to better understand, manage, and treat patients who present with arbovirus infection.

## Supplementary Material

Patient presentation of AMS at 24 hour after admission. Physician performance of the Brudzinski maneuver elicits a positive response. Of note is nucchal rigidity and the myoclonic tremor of the upper extremities.

## Figures and Tables

**Table 1:** Results of laboratory testing on the patient cerebrospinal fluid.

Test	Result	Reference ranges
Color	Clear	Clear/colorless
WBC (per *μ*L)	530	0–5
Neutrophil (%)	76	0–5
Lymphocyte (%)	10	0–5
Monocyte (%)	14	0–5
RBC (per *μ*L)	15	0
Glucose (mg/dL)	91	50–80 mg/dL
Protein (mg/dL)	154	15–60 mg/dL
Gram stain/culture	No organisms	No organisms
VDRL culture	No organisms	No organisms
Fungal culture	No organisms	No organisms
HSV-1/2 PCR	Negative	Negative
Crypto Ag	Negative	Negative
Cocci IgG/IgM	Negative	Negative
WNV IgG	**1.86**	<1.30
WNV IgM	**4.43**	<0.90

VDRL indicates venereal disease research laboratory; HSV, Herpes simplex virus; Crypto, *Cryptococcus*; Ag, antigen; cocci, coccidiomycosis; WNV, West Nile Virus. Reference ranges for WNV ELISA titer are values IgG < 1.30, IgM < 0.90.
